# Supplementary material for: In silico clinical trials of BiTE expression by oncolytic viruses reveal the impact of patient heterogeneity on dosage protocol
Source: PLoS Comput Biol. 2026 Jul 30;22(7):e1014533. doi: 10.1371/journal.pcbi.1014533 (PMC13446748; doi:10.1371/journal.pcbi.1014533)
Supplement: Supplementary file 2 [file pcbi.1014533.s010.pdf]

In silico clinical trials of BiTE expression by oncolytic viruses reveal the impact of patient heterogeneity on dosage protocol

by
Jenner et al.

Overall Comments

The mathematical modeling work done in this manuscript tackles one of the essential problems in immunotherapy, where the efficacy of combining Bispecific T-cell engagers (BiTEs) and oncolytic virotherapy remain largely undefined, particularly when applied to a heterogeneous human population. Global sensitivity analysis of the mathematical model revealed some intriguing threshold parameters, namely the immune-related parameters T cell killing rate (k), basal T cell killing constant (ϵ), and stimulation of APCs (s) had a negative correlation with the tumor size, which implies that if these parameters are increase, then tumor burden will be reduced. The introduction is very well written and informative.

The simulations are very clear with interesting virtual experiments "mimicking" real patient dynamics. The study is theoretically in mature, however, it is grounded and based on a simple compartmental model which is validated on both in vitro and in vivo studies using oncolytic measles virus (MV) encoding and not encoding BiTEs, as well as some Phase 2 clinical data using talimogene laherparepvec (T-VEC). The model herein provides useful insights and in my opinion should be accepted for publication, with minor comments to be addressed below.

I have minor comments that may improve the quality of the work.

1) On page 8, line 221, the parameter d_T = 0.35 has no units, though all parameters are listed with their units in Table 2. I think the units should be restated here as has been done everywhere in the paper (e.g., from line 223-224, "dead cell clearance rate of d_D = 4.8/day [72]. For fitting the in vivo data: B(0) = 2.17µg, ...").
2) On page 8, from line 208, the global sensitivity analysis done here is suitable and adequately explained. In future follow up work to the present study, I suggest that authors can try one or more of other advanced variance-based methods of the global sensitivity analyses such as eFAST or MeFAST or Sobol) to get a full picture of model sensitivity to baseline parameters.
3) On page 9, in Table 2, in the description "stimulation of APCs", the abbreviation "APCs" is not defined anywhere in previous sections. Thus, it need to be clearly defined.

Summary

The paper is well written and the figures easy to follow. The methods and model descriptions are thorough, and the analysis very clearly described. In general, the paper is generally well written with only a few points where the authors could consider adding more details or be aware of alternative explanations. While the conclusions drawn in this study are only hypothetical (since they are based on virtual cohorts), they pinpoint and highlight certain important parameters, identified by reliable sensitivity method using Pearson Correlation Coefficient (PCC). The mathematical formulation is strong and provide sufficient explanation. The ideas or goals of this study are of paramount significance, though there few simulations that can be more interesting if they are compared with findings from other robust sensitivity methods such as the extended Fourier Amplitude Sensitivity Testing (eFAST). The authors show, convincingly (at least in the model simulations), that patient-derived xenograft models with tumor cells of human origin showed extended BiTE expression compared to the murine tumor model. In future, it would be interesting to see how the model developed in this study fits the human clinical trial data for MV-BiTE. Overall, I think this is an excellent paper, timely for publication in PLOS Computational Biology, which should be accepted after minor revisions of the points raised above.
