# Supplementary material for: In silico clinical trials of BiTE expression by oncolytic viruses reveal the impact of patient heterogeneity on dosage protocol
Source: PLoS Comput Biol. 2026 Jul 30;22(7):e1014533. doi: 10.1371/journal.pcbi.1014533 (PMC13446748; doi:10.1371/journal.pcbi.1014533)
Supplement: Supplementary file 3 [file pcbi.1014533.s012.pdf]

We thank the Editors and Reviewers for their feedback. All of the comments were very helpful and have been addressed below, with changes highlighted in the manuscript.

Reviewer 1

The manuscript addresses the emerging role of bispecific T-cell engagers (BiTEs) and their integration within oncolytic viral platforms in clinical practice. The study explores a novel therapeutic strategy in which BiTEs are encoded within oncolytic measles virus vectors (MV-BiTE), enabling localized production of immune-activating agents directly within tumour sites. This approach aims to enhance T-cell-mediated cytotoxicity against cancer cells. Recognising the challenge of translating preclinical efficacy into heterogeneous human populations, the authors develop an in silico clinical trial based on a system of ordinary differential equations. By incorporating inter-patient variability derived from tumour volume data and baseline measurements from a Phase II clinical trial, the model provides predictive insights into treatment response. Notably, the study identifies effector T-cell killing rates and BiTE pharmacokinetics as primary drivers of heterogeneity and suggests that modified dosing regimens may improve outcomes in non-responding patients. Overall, the work highlights the potential of in silico trials to inform clinical translation and optimise therapeutic design.

The authors are to be commended for the quality of the work, which is already presented in a form very close to publication standard. The manuscript is clearly structured, with a logical organisation into well-defined sections that facilitate readability. The exposition is precise and accessible, and the supplementary material is thoughtfully organised, allowing the reader to efficiently locate methodological details and supporting information. Furthermore, the mathematical framework is, for the most part, well-suited to the biological problem under investigation. The study demonstrates a high level of biological relevance and, importantly, embodies what should be the role of applied mathematics in biomedical research, actively contributing to hypothesis generation and potentially informing clinical decision-making.

We thank Reviewer 1 for their feedback and time spent reviewing our manuscript. We have responded to all queries and have made appropriate changes in the manuscript.

That said, I would like to raise several minor points that, if addressed, could further strengthen the manuscript:

- Limited characterisation of T-cell dynamics (activation, expansion, exhaustion)
The current model appears to simplify T-cell behaviour by focusing primarily on cytotoxic activity, without explicitly accounting for key immunological processes such as activation thresholds, clonal expansion, and functional exhaustion. These processes are known to play a critical role in shaping the efficacy and sustainability of anti-tumour immune responses, particularly in the context of chronic antigen exposure typical of cancer. The omission of such mechanisms may limit the biological interpretability and predictive reliability of the model. For instance, T-cell exhaustion could significantly alter long-term treatment outcomes, especially under repeated dosing schedules. Expanding the model to include additional compartments or state variables representing these processes, or, at minimum, discussing their potential impact and justification for their exclusion, would enhance the robustness of the biological conclusions.

We thank the reviewer for this excellent comment highlighting the need for future expansion of the mathematical model towards including additional characterisations of T cell responses and repertoire. We agree that T cell exhaustion will influence the development of long-lasting anti-tumour responses. This is supported by both our own preclinical data (Speck et al. 2018, supplemental Fig. S10) and clinical evidence (van de Donk et al. 2024). We do believe, however, that the current implementation of the model does reflect the limited dataset to an acceptable degree, and that activation thresholds and clonal expansion might be better reflected by approaches such as agent-based modeling. We have therefore added a paragraph discussing this limitation of our model as well as giving an outlook for ongoing work in this regard.

> Pg 20: *"Furthermore, a wide range of biological processes is involved in anti-tumour T cell effects, including activation via the endogenous T cell receptor and/or bispecific T cell engagers, replication of individual T cell clones, stimulation and inhibition by cytokines and other mechanisms, and T cell exhaustion. While our model captures rates of T cell accumulation, decay, and baseline as well as BiTE-induced tumor cell killing, it does not explicitly account for T cell exhaustion, which is a common mechanism found in cancer and, more specifically, in both OV and BiTE therapies. Various concepts such as OV-encoded immune checkpoint inhibitors or BiTE treatment-free intervals have been devised to address this [85-88]. In a future version of the model, we aim to implement T cell exhaustion on the basis of time-resolved functional T cell characterisation to better reflect cases of treatment failure under continuous BiTE release by OV-infected cells. This can subsequently be used to predict optimal combination or dosing strategies for long-term anti-tumour efficacy with specific focus on functional exhaustion of T cells."*

- Use of MATLAB and implications for reproducibility

The implementation of the computational framework in MATLAB, while standard in many applied mathematics contexts, may limit accessibility and reproducibility, particularly for researchers without access to proprietary software. Given the increasing emphasis on open science, the use of open-source alternatives (such as Python-based ecosystems or Julia) could facilitate broader validation and reuse of the model. At the very least, the authors might consider providing detailed documentation or exploring options for sharing code in a more accessible format to improve reproducibility.

We appreciate the reviewer's suggestion here and agree that accessibility and reproducibility are very important. To help facilitate broader use of our modelling, we have updated the GitHub with a more detailed read me file, and examples of additional code from our modelling: https://github.com/AdrianneJennerQUT/insilico-clinical-trial-MV-BiTEs/tree/main. We also agree with the reviewer's point about accessibility being limited by our use of MATLAB. We have now provided a Julia equivalent code for solving our model in the GitHub as well.

-Deterministic choice of initial conditions

The selection of initial conditions (e.g., line 258 and related sections) appears somewhat deterministic, which may not fully reflect the variability inherent in biological systems and patient populations. Since the study explicitly aims to capture inter-individual heterogeneity, a more thorough justification of these choices would be valuable. In particular, it would be useful to clarify whether sensitivity analyses were conducted with respect to initial conditions, and how robust the model predictions are to such variations. Incorporating stochastic elements or distributions for initial states could further align the modelling framework with the variability observed in clinical settings.

The reviewer makes an excellent suggestion about investigating the effects of initial condition heterogeneity on the resulting tumour burden. We have now conducted an additional analysis (see Figure 8B-C) where we simulated 400 unique initial tumour sizes and compared the resulting change from baseline for our virtual cohort to the true clinical cohort. We found heterogeneity in the initial conditions were unable to reproduce the spread of the true clinical cohort data, suggesting that patient tumour/immune characteristics must vary for the data to be captured. See additional text added below, new figures in Figure 8B-C and additional methods in the SI.

> Pg 9-11: "*Previous work by Dessinioti [73] et al. reported a median melanoma diameter of 13mm, with quartiles 9 - 20mm, and 10.6% of tumours below 6mm. Using these statistical summaries, we parameterised a gamma distribution and obtained a shape and scale parameter of 3.09 and 4.91 respectively (see Supplementary Information)*
>
> *.... For the first virtual cohort investigation of the clinical trial measurements, we sampled 400 tumour diameters from the parameterised gamma distribution and converted them to cell counts which were used as initial conditions for the model. Following this, we fixed the initial cell count to the median tumour diameter, i.e. 13mm, and obtain virtual patients using Method (2) described above."*

> Pg 16: *"We first examined whether heterogeneity in the initial tumour burden could explain the change from baseline (CFB) measurements from the clinical trial. To do this, we parameterised a gamma distribution using summary statistics reported by Dessinioti et al. [73] for the diameter of melanomas in 537 cases (see Methods and Supplementary Information for details). We then sampled 400 virtual patient initial tumour diameters (Fig 8B) and converted these to initial cell counts to feed into the model. The resulting CFB predicted from the model for heterogeneous initial tumour sizes was unable to capture the range of the clinical trial measurements (Fig 8C). This suggests that changes to parameters, representing patient-specific characteristics, are needed to capture clinical outcomes."*

-Potential inconsistency in dosing interpretation

The manuscript states that "lower-dosage injections given more frequently were predicted to reduce the tumour size most significantly," and that "more frequent injections were optimal." However, this is followed by the claim that "having periods of time between injections is more likely to be successful than giving large bolus injections close together." This latter statement appears, at least superficially, to introduce some ambiguity. If frequent administration is optimal, it would be helpful to clarify what is meant by "periods of time between injections," and how this differs from the concept of frequent dosing. Is the key factor the avoidance of high-dose clustering, rather than frequency per se? A more precise formulation of this point would improve clarity and prevent potential misinterpretation

We thank the reviewer for pointing this out. Our original description of the results on dosing was not clear. We have now revised this paragraph to clearly summarise our results: the most effective protocol is an initial dose with a wait before the second dose and then frequent, smaller dosages thereafter. We have updated the paragraph in the discussion to make this point clearer:

> Pg 18-19: *"We found consistently throughout our simulations that lower-dosage injections given more frequently were predicted to reduce the tumour size most significantly. In both the in silico clinical trials based on the in vivo tumour growth and the Phase II clinical trial data, we saw that more frequent injections were optimal. Furthermore, we found that an initial dosage followed by a short delay in the following booster dosage (3 weeks) followed by frequent small doses was most effective. This suggests that once the treatment effect has time to establish after the first dosage, frequent small dosages are more effective than large bolus dosages given far apart. However, in experimental and clinical practice, this might pose a challenge due to tumour growth in the intervals between injections and, therefore, needs to be carefully evaluated in a well-designed animal experiment beforehand."*

In summary, this is a strong and well-executed study that makes a meaningful contribution to the field of mathematical oncology and immunotherapy modelling. Addressing the points above would further enhance its clarity, reproducibility, and biological depth.

Reviewer 2

Overall Comments

The mathematical modeling work done in this manuscript tackles one of the essential problems in immunotherapy, where the efficacy of combining Bispecific T-cell engagers (BiTEs) and oncolytic virotherapy remain largely undefined, particularly when applied to a heterogeneous human population. Global sensitivity analysis of the mathematical model revealed some intriguing threshold parameters, namely the immune-related parameters T cell killing rate (k), basal T cell killing constant (ϵ), and stimulation of APCs (s) had a negative correlation with the tumor size, which implies that if these parameters are increase, then tumor burden will be reduced. The introduction is very well written and informative. The simulations are very clear with interesting virtual experiments "mimicking" real patient dynamics. The study is theoretically in mature, however, it is grounded and based on a simple compartmental model which is validated on both in vitro and in vivo studies using oncolytic measles virus (MV) encoding and not encoding BiTEs, as well as some Phase 2 clinical data using talimogene laherparepvec (T-VEC). The model herein provides useful insights and in my opinion should be accepted for publication, with minor comments to be addressed below. I have minor comments that may improve the quality of the work.

1) On page 8, line 221, the parameter d_T = 0.35 has no units, though all parameters are listed with their units in Table 2. I think the units should be restated here as has been done everywhere in the paper (e.g., from line 223-224, "dead cell clearance rate of d_D = 4.8/day [72]. For fitting the in vivo data: B(0) = 2.17μg, ...").

We agree with the reviewer and have updated the text accordingly.

2) On page 8, from line 208, the global sensitivity analysis done here is suitable and adequately explained. In future follow up work to the present study, I suggest that authors can try one or more of other advanced variance-based methods of the global sensitivity analyses such as eFAST or MeFAST or Sobol) to get a full picture of model sensitivity to baseline parameters.

We thank the reviewer for this suggestion and agree we will definitely try this in future sensitivity analysis. We have added a note to this method in the main text:

> Pg 8: *Other sensitivity analysis methods, such as eFAST, MeFAST or Sobol, would also have been appropriate here"*

3) On page 9, in Table 2, in the description "stimulation of APCs", the abbreviation "APCs" is not defined anywhere in previous sections. Thus, it need to be clearly defined.

We thank the reviewer for catching this error, this was a typo in the table and it has now been corrected.

Summary

The paper is well written and the figures easy to follow. The methods and model descriptions are thorough, and the analysis very clearly described. In general, the paper is generally well written with only a few points where the authors could consider adding more details or be aware of alternative explanations. While the conclusions drawn in this study are only hypothetical (since they are based on

virtual cohorts), they pinpoint and highlight certain important parameters, identified by reliable sensitivity method using Pearson Correlation Coefficient (PCC). The mathematical formulation is strong and provide sufficient explanation. The ideas or goals of this study are of paramount significance, though there few simulations that can be more interesting if they are compared with findings from other robust sensitivity methods such as the extended Fourier Amplitude Sensitivity Testing (eFAST). The authors show, convincingly (at least in the model simulations), that patient-derived xenograft models with tumor cells of human origin showed extended BiTE expression compared to the murine tumor model. In future, it would be interesting to see how the model developed in this study fits the human clinical trial data for MV-BiTE. Overall, I think this is an excellent paper, timely for publication in PLOS Computational Biology, which should be accepted after minor revisions of the points raised above.
