# Supplementary material for: In silico clinical trials of BiTE expression by oncolytic viruses reveal the impact of patient heterogeneity on dosage protocol
Source: PLoS Comput Biol. 2026 Jul 30;22(7):e1014533. doi: 10.1371/journal.pcbi.1014533 (PMC13446748; doi:10.1371/journal.pcbi.1014533)
Supplement: Peer review history

# Peer review: Decision Letter 0

**DOI:** 10.1371/journal.pcbi.1014533.r001

**Authors:** Amber Smith, David Basanta Gutierrez

17 May 2026

PCOMPBIOL-D-26-00091

In silico clinical trials of BiTE expression by oncolytic viruses reveal the impact of patient heterogeneity on dosage protocol

PLOS Computational Biology

Dear Dr. Jenner,

Thank you for submitting your manuscript to PLOS Computational Biology. After careful consideration, we feel that it has merit but does not fully meet PLOS Computational Biology's publication criteria as it currently stands. Therefore, we invite you to submit a revised version of the manuscript that addresses the points raised during the review process.

Please submit your revised manuscript by Jul 17 2026 11:59PM. If you will need more time than this to complete your revisions, please reply to this message or contact the journal office at ploscompbiol@plos.org. When you're ready to submit your revision, log on to https://www.editorialmanager.com/pcompbiol/ and select the 'Submissions Needing Revision' folder to locate your manuscript file.

Please include the following items when submitting your revised manuscript:

* A letter that responds to each point raised by the editor and reviewer(s). You should upload this letter as a separate file labeled 'Response to Reviewers'. This file does not need to include responses to formatting updates and technical items listed in the 'Journal Requirements' section below.

* A marked-up copy of your manuscript that highlights changes made to the original version. You should upload this as a separate file labeled 'Revised Manuscript with Track Changes'.

* An unmarked version of your revised paper without tracked changes. You should upload this as a separate file labeled 'Manuscript'.

If you would like to make changes to your financial disclosure, competing interests statement, or data availability statement, please make these updates within the submission form at the time of resubmission. Guidelines for resubmitting your figure files are available below the reviewer comments at the end of this letter.

We look forward to receiving your revised manuscript.

Kind regards,

David Basanta Gutierrez

Academic Editor

PLOS Computational Biology

Dominik Wodarz

Section Editor

PLOS Computational Biology

**Journal Requirements:**

If the reviewer comments include a recommendation to cite specific previously published works, please review and evaluate these publications to determine whether they are relevant and should be cited. There is no requirement to cite these works unless the editor has indicated otherwise.

1) Please ensure that the CRediT author contributions listed for every co-author are completed accurately and in full.

At this stage, the following Authors/Authors require contributions: Adrianne L Jenner, Robyn P Araujo, Christine E Engeland, Johannes P. W. Heidbuechel, Noa Levi, and Guy Ungerechts. Please ensure that the full contributions of each author are acknowledged in the "Add/Edit/Remove Authors" section of our submission form.

The list of CRediT author contributions may be found here: https://journals.plos.org/ploscompbiol/s/authorship#loc-author-contributions

2) We ask that a manuscript source file is provided at Revision. Please upload your manuscript file as a .doc, .docx, .rtf or .tex. If you are providing a .tex file, please upload it under the item type u2018LaTeX Source Fileu2019 and leave your .pdf version as the item type u2018Manuscriptu2019.

3) Your manuscript's sections are not in the correct order.  Please amend to the following order: Abstract, Introduction, Results, Discussion, and Methods

4) Please upload all main figures as separate Figure files in .tif or .eps format. For more information about how to convert and format your figure files please see our guidelines:

https://journals.plos.org/ploscompbiol/s/figures

5) We note that  1, and 2 are  created through BioRender. Please confirm that you hold a Premium account and provide a pdf copy of the CC BY 4.0 Licence as provided by BioRender. For instructions on how to generate a CC BY 4.0 license for your figure, please see the guidelines here: https://help.biorender.com/hc/en-gb/articles/21282341238045-Publishing-in-open-access-resources

Please ensure the figure legend contains appropriate attribution text & a link to biorender.com

If you are using the free assets from BioRender, we are unable to publish these images as they are licenced under a stricter licence than CC BY 4.0. In this case we ask you to remove the BioRender images and replace them with open source alternatives.

See these open source resources you may use to replace images / clip-art:

- https://bioart.niaid.nih.gov/

- https://bioicons.com/

- https://healthicons.org/

- https://scidraw.io/

- https://reactome.org/icon-lib

- https://www.phylopic.org/images

- https://journals.plos.org/plosbiology/article?id=10.1371/journal.pbio.3002395

6) Some material included in your submission may be copyrighted. According to PLOS’s copyright policy, authors who use figures or other material (e.g., graphics, clipart, maps) from another author or copyright holder must demonstrate or obtain permission to publish this material under the Creative Commons Attribution 4.0 International (CC BY 4.0) License used by PLOS journals. Please closely review the details of PLOS’s copyright requirements here: PLOS Licenses and Copyright. If you need to request permissions from a copyright holder, you may use PLOS's Copyright Content Permission form.

Please respond directly to this email and provide any known details concerning your material's license terms and permissions required for reuse, even if you have not yet obtained copyright permissions or are unsure of your material's copyright compatibility. Once you have responded and addressed all other outstanding technical requirements, you may resubmit your manuscript within Editorial Manager.

Potential Copyright Issues:

i) Figures 3, 4A, 5A, and 7A. Please confirm whether you drew the images / clip-art within the figure panels by hand. If you did not draw the images, please provide (a) a link to the source of the images or icons and their license / terms of use; or (b) written permission from the copyright holder to publish the images or icons under our CC BY 4.0 license. Alternatively, you may replace the images with open source alternatives. See these open source resources you may use to replace images / clip-art:

- https://commons.wikimedia.org

- https://openclipart.org/.

7) Please amend your detailed Financial Disclosure statement. This is published with the article. It must therefore be completed in full sentences and contain the exact wording you wish to be published.

1) State what role the funders took in the study. If the funders had no role in your study, please state: "The funders had no role in study design, data collection and analysis, decision to publish, or preparation of the manuscript."

2) If any authors received a salary from any of your funders, please state which authors and which funders..

If you did not receive any funding for this study, please simply state: u201cThe authors received no specific funding for this work.u201d

8)  Please ensure that the funders and grant numbers match between the Financial Disclosure field and the Funding Information tab in your submission form. Note that the funders must be provided in the same order in both places as well.

9) Please send a completed 'Competing Interests' statement, including any COIs declared by your co-authors. If you have no competing interests to declare, please state "The authors have declared that no competing interests exist". Otherwise please declare all competing interests beginning with the statement "I have read the journal's policy and the authors of this manuscript have the following competing interests"

**Reviewers' comments:**

Reviewer's Responses to Questions

**Comments to the Authors:**

**Please note here if the review is uploaded as an attachment.**

Reviewer #1: The manuscript addresses the emerging role of bispecific T-cell engagers (BiTEs) and their integration within oncolytic viral platforms in clinical practice. The study explores a novel therapeutic strategy in which BiTEs are encoded within oncolytic measles virus vectors (MV-BiTE), enabling localized production of immune-activating agents directly within tumour sites. This approach aims to enhance T-cell-mediated cytotoxicity against cancer cells. Recognising the challenge of translating preclinical efficacy into heterogeneous human populations, the authors develop an in silico clinical trial based on a system of ordinary differential equations. By incorporating inter-patient variability derived from tumour volume data and baseline measurements from a Phase II clinical trial, the model provides predictive insights into treatment response. Notably, the study identifies effector T-cell killing rates and BiTE pharmacokinetics as primary drivers of heterogeneity and suggests that modified dosing regimens may improve outcomes in non-responding patients. Overall, the work highlights the potential of in silico trials to inform clinical translation and optimise therapeutic design.

The authors are to be commended for the quality of the work, which is already presented in a form very close to publication standard. The manuscript is clearly structured, with a logical organisation into well-defined sections that facilitate readability. The exposition is precise and accessible, and the supplementary material is व्यव thoughtfully organised, allowing the reader to efficiently locate methodological details and supporting information. Furthermore, the mathematical framework is, for the most part, well-suited to the biological problem under investigation. The study demonstrates a high level of biological relevance and, importantly, embodies what should be the role of applied mathematics in biomedical research, actively contributing to hypothesis generation and potentially informing clinical decision-making.

That said, I would like to raise several minor points that, if addressed, could further strengthen the manuscript:

- Limited characterisation of T-cell dynamics (activation, expansion, exhaustion)

The current model appears to simplify T-cell behaviour by focusing primarily on cytotoxic activity, without explicitly accounting for key immunological processes such as activation thresholds, clonal expansion, and functional exhaustion. These processes are known to play a critical role in shaping the efficacy and sustainability of anti-tumour immune responses, particularly in the context of chronic antigen exposure typical of cancer. The omission of such mechanisms may limit the biological interpretability and predictive reliability of the model. For instance, T-cell exhaustion could significantly alter long-term treatment outcomes, especially under repeated dosing schedules. Expanding the model to include additional compartments or state variables representing these processes, or, at minimum, discussing their potential impact and justification for their exclusion, would enhance the robustness of the biological conclusions.

- Use of MATLAB and implications for reproducibility

The implementation of the computational framework in MATLAB, while standard in many applied mathematics contexts, may limit accessibility and reproducibility, particularly for researchers without access to proprietary software. Given the increasing emphasis on open science, the use of open-source alternatives (such as Python-based ecosystems or Julia) could facilitate broader validation and reuse of the model. At the very least, the authors might consider providing detailed documentation or exploring options for sharing code in a more accessible format to improve reproducibility.

-Deterministic choice of initial conditions

The selection of initial conditions (e.g., line 258 and related sections) appears somewhat deterministic, which may not fully reflect the variability inherent in biological systems and patient populations. Since the study explicitly aims to capture inter-individual heterogeneity, a more thorough justification of these choices would be valuable. In particular, it would be useful to clarify whether sensitivity analyses were conducted with respect to initial conditions, and how robust the model predictions are to such variations. Incorporating stochastic elements or distributions for initial states could further align the modelling framework with the variability observed in clinical settings.

-Potential inconsistency in dosing interpretation

The manuscript states that “lower-dosage injections given more frequently were predicted to reduce the tumour size most significantly,” and that “more frequent injections were optimal.” However, this is followed by the claim that “having periods of time between injections is more likely to be successful than giving large bolus injections close together.” This latter statement appears, at least superficially, to introduce some ambiguity. If frequent administration is optimal, it would be helpful to clarify what is meant by “periods of time between injections,” and how this differs from the concept of frequent dosing. Is the key factor the avoidance of high-dose clustering, rather than frequency per se? A more precise formulation of this point would improve clarity and prevent potential misinterpretation.

In summary, this is a strong and well-executed study that makes a meaningful contribution to the field of mathematical oncology and immunotherapy modelling. Addressing the points above would further enhance its clarity, reproducibility, and biological depth.

Reviewer #2: Please see the attached document.

**********

**Have the authors made all data and (if applicable) computational code underlying the findings in their manuscript fully available?**

The PLOS Data policy requires authors to make all data and code underlying the findings described in their manuscript fully available without restriction, with rare exception (please refer to the Data Availability Statement in the manuscript PDF file). The data and code should be provided as part of the manuscript or its supporting information, or deposited to a public repository. For example, in addition to summary statistics, the data points behind means, medians and variance measures should be available. If there are restrictions on publicly sharing data or code —e.g. participant privacy or use of data from a third party—those must be specified.

Reviewer #1: Yes

Reviewer #2: Yes

**********

PLOS authors have the option to publish the peer review history of their article (what does this mean?). If published, this will include your full peer review and any attached files.

If you choose “no”, your identity will remain anonymous but your review may still be made public.

**Do you want your identity to be public for this peer review?**  For information about this choice, including consent withdrawal, please see our Privacy Policy.

Reviewer #1: No

Reviewer #2: **Yes:**  Khaphetsi Joseph Mahasa

[NOTE: If reviewer comments were submitted as an attachment file, they will be attached to this email and accessible via the submission site. Please log into your account, locate the manuscript record, and check for the action link "View Attachments". If this link does not appear, there are no attachment files.]

**Figure resubmission:**

-->While revising your submission, we strongly recommend that you use PLOS’s NAAS tool (https://ngplosjournals.pagemajik.ai/artanalysis) to test your figure files. NAAS can convert your figure files to the TIFF file type and meet basic requirements (such as print size, resolution), or provide you with a report on issues that do not meet our requirements and that NAAS cannot fix.-->

After uploading your figures to PLOS’s NAAS tool - https://ngplosjournals.pagemajik.ai/artanalysis, NAAS will process the files provided and display the results in the "Uploaded Files" section of the page as the processing is complete. If the uploaded figures meet our requirements (or NAAS is able to fix the files to meet our requirements), the figure will be marked as "fixed" above. If NAAS is unable to fix the files, a red "failed" label will appear above. When NAAS has confirmed that the figure files meet our requirements, please download the file via the download option, and include these NAAS processed figure files when submitting your revised manuscript.

**Reproducibility:**

To enhance the reproducibility of your results, we recommend that authors of applicable studies deposit laboratory protocols in protocols.io, where a protocol can be assigned its own identifier (DOI) such that it can be cited independently in the future. Additionally, PLOS ONE offers an option to publish peer-reviewed clinical study protocols. Read more information on sharing protocols at https://plos.org/protocols?utm_medium=editorial-email&utm_source=authorletters&utm_campaign=protocols

Attachment Submitted filename: PCOMPBIOL-D-26-00091_reviewer_Mahasa.pdf

# Author comment: Author response to Decision Letter 1

**DOI:** 10.1371/journal.pcbi.1014533.r002

23 Jun 2026

Attachment Submitted filename: Reviewer comments_collated.pdf

# Editor report: Decision Letter 1

**DOI:** 10.1371/journal.pcbi.1014533.r003

**Authors:** Amber Smith, David Basanta Gutierrez

5 Jul 2026

Dear Dr. Jenner,

We are pleased to inform you that your manuscript 'In silico clinical trials of BiTE expression by oncolytic viruses reveal the impact of patient heterogeneity on dosage protocol' has been provisionally accepted for publication in PLOS Computational Biology.

Before your manuscript can be formally accepted you will need to complete some formatting changes, which you will receive in a follow up email. A member of our team will be in touch with a set of requests.

Please note that your manuscript will not be scheduled for publication until you have made the required changes, so a swift response is appreciated.

IMPORTANT: The editorial review process is now complete. PLOS will only permit corrections to spelling, formatting or significant scientific errors from this point onwards. Requests for major changes, or any which affect the scientific understanding of your work, will cause delays to the publication date of your manuscript.

Should you, your institution's press office or the journal office choose to press release your paper, you will automatically be opted out of early publication. We ask that you notify us now if you or your institution is planning to press release the article. All press must be co-ordinated with PLOS.

Thank you again for supporting Open Access publishing; we are looking forward to publishing your work in PLOS Computational Biology.

Best regards,

David Basanta Gutierrez

Academic Editor

PLOS Computational Biology

Amber Smith

Section Editor

PLOS Computational Biology

***********************************************************

# Editor report: Acceptance letter

**DOI:** 10.1371/journal.pcbi.1014533.r004

**Authors:** Amber Smith, David Basanta Gutierrez

PCOMPBIOL-D-26-00091R1

In silico clinical trials of BiTE expression by oncolytic viruses reveal the impact of patient heterogeneity on dosage protocol

Dear Dr Jenner,

I am pleased to inform you that your manuscript has been formally accepted for publication in PLOS Computational Biology. Your manuscript is now with our production department and you will be notified of the publication date in due course.

The corresponding author will soon be receiving a typeset proof for review, to ensure errors have not been introduced during production. Please review the PDF proof of your manuscript carefully, as this is the last chance to correct any errors. Please note that major changes, or those which affect the scientific understanding of the work, will likely cause delays to the publication date of your manuscript.

Soon after your final files are uploaded, unless you have opted out, the early version of your manuscript will be published online. The date of the early version will be your article's publication date. The final article will be published to the same URL, and all versions of the paper will be accessible to readers.

For Research, Software, and Methods articles, you will receive an invoice from PLOS for your publication fee after your manuscript has reached the completed accept phase. If you receive an email requesting payment before acceptance or for any other service, this may be a phishing scheme. Learn how to identify phishing emails and protect your accounts at https://explore.plos.org/phishing.

Thank you again for supporting PLOS Computational Biology and open-access publishing. We are looking forward to publishing your work!

With kind regards,

Sharmila Kamatchi

PLOS Computational Biology | Carlyle House, Carlyle Road, Cambridge CB4 3DN | United Kingdom ploscompbiol@plos.org | Phone +44 (0) 1223-442824 | ploscompbiol.org | @PLOSCompBiol
